# Computer-assisted implant placement and full-arch immediate loading with digitally prefabricated provisional prostheses without cast: a prospective pilot cohort study

**DOI:** 10.1186/s40729-021-00369-0

**Published:** 2021-09-06

**Authors:** Nikolay Makarov, Giorgio Pompa, Piero Papi

**Affiliations:** 1 Department of Oral and Maxillo-Facial Sciences, “Sapienza” University of Rome, Via Caserta, 6, 00161 Rome, Italy; 2 Oral Surgery Unit, Department of Oral and Maxillo-Facial Sciences, “Sapienza” University of Rome, Rome, Italy

**Keywords:** CAD-CAM, Surgery, Computer-Assisted, Immediate dental implant loading, Dental implants, Printing, Three-Dimensional

## Abstract

**Background:**

Immediate loading of implant-supported full-arch rehabilitations has become routine practice when treating edentulous patients. The combination of static computer-aided implant surgery (s-CAIS) and digital prosthetic workflow could eliminate several treatment steps and facilitate prostheses delivery.

The aim of this study is to evaluate the 1-year results of digitally prefabricated polymethyl methacrylate (PMMA) provisional prostheses without a cast for full-arch computer-assisted immediate loading.

**Materials and methods:**

A digital pre-operative treatment planning was realized for all patients: dental implants and screw-retained abutments were selected in the planning software and two surgical templates were fabricated for each patient. The first template was mucosa or teeth-supported to drill the holes for fixating pins, while the second template was placed after raising a full-thickness flap and was supported by pins as well as soft or hard tissue distal support. Furthermore, based on the surgical planning, interim prostheses were digitally designed and milled of PMMA resin blocks with subsequent pink resin veneering. Osteotomies and implant placement were performed through the surgical guides and all implants were immediately loaded with prefabricated full-arch interim prostheses directly connected to titanium copings with a flowable resin.

**Results:**

A total of 55 dental implants were placed in ten patients. In all cases, interim prostheses allowed the insertion of titanium copings without the need of access hole enlargement or adaptation. All the prostheses had 1 year of functional loading to simulate the long provisional phase. No screw loosening occurred at the first removal of the prostheses after implant osseointegration. No fracture occurred during the whole period. After 1 year, the mean marginal bone loss level was 0.37 ± 0.06 mm, while the implant survival rate was 98.18% (n=54/55), with just one implant failing but not affecting final prosthesis delivery to the patient.

**Conclusions:**

Within the limitations of the present study, the authors concluded that digitally prefabricated provisional prostheses for full-arch immediate loading with s-CAIS could be a valid alternative treatment modality. Milled PMMA restorations proved to be durable enough during the long provisional phase, without prosthetic complications.

## Background

Nowadays, immediate loading of implant-supported full-arch rehabilitation has become a routine practice when treating edentulous patients, giving comparable results to conventional and early loading protocols, improving patient acceptance and comfort [1, 2]. The conventional procedure includes freehand implant placement, impression taking, and prosthesis delivery within 1 week after surgery [3]. However, this time frame might be too long for either the dentist or the patient. According to recent articles, static computer-aided surgery (s-CAIS) has shown an acceptable level of accuracy [4–7]. However, interim prostheses production process might be affected by working cast and impression contraction and deformation [8]. There are several options to deliver an interim prosthesis: the conversion of a pre-existent complete denture [9, 10] or the fabrication of a new prosthesis. According to Lin et al. [11], the most common complications associated with an interim full-arch fixed acrylic resin prosthesis are the fracture of the prosthetic structure and fracture of the veneering material, while based on Crespi et al.’s [12] results, immediate restorations had the same clinical outcome regardless of whether they were reinforced with a metal framework. Furthermore, a greater fracture rate has been reported when converting an existing denture, due to the lack of strength caused by the creation of the access holes [13, 14]. Digital workflow could eliminate several treatment steps, such as impression taking, reducing the risk of fracture, and the need for a reinforcement of the interim prostheses, due to the use of more durable CAD/CAM materials, such as milled polymethyl methacrylate (PMMA) [15, 16]. Just a few studies have documented prosthesis fabrication before implant surgery [17–20]. In the present article, the authors would like to discuss an alternative digital workflow for implant-supported full-arch rehabilitations. The aims of this study are to evaluate the 1-year results of digitally prefabricated PMMA provisional prostheses for full-arch computer-assisted immediate loading.

## Materials and methods

To address the research purpose, the authors designed and implemented this prospective pilot cohort study, conducted at the University clinic and approved by the local Institution Review Board of the Department of Oral and Maxillo-Facial Sciences at “Sapienza” University of Rome. The study sample was composed by patients presenting at the university department for implant treatment of complete edentulism. In order to be included in the study, patients had to meet the following inclusion and exclusion criteria: fully edentulous jaws or failing dentitions, good oral hygiene (FMPS and FMBS < 25%), absence of uncontrolled systemic diseases, non-smokers (< 10 cigarettes/day), non-pregnancy or lactation, and no signs of local inflammation. All patients included signed the informed consent forms according to the latest edition of the World Medical Association’s Declaration of Helsinki and declared their commitment to participate for the full duration of the study.

**Fig. 1:**
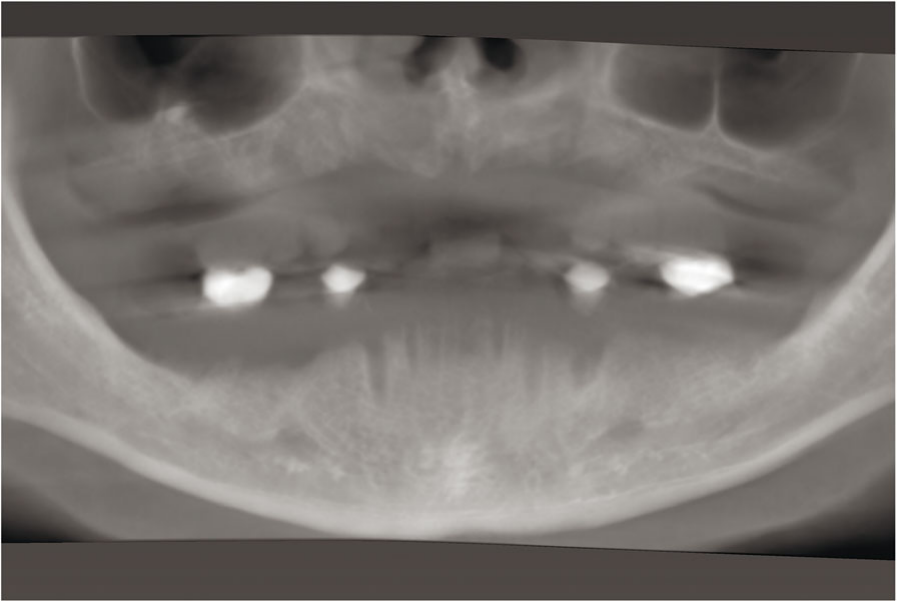
Pre-operative CBCT with radiographic template and radiopaque teeth

**Fig. 2:**
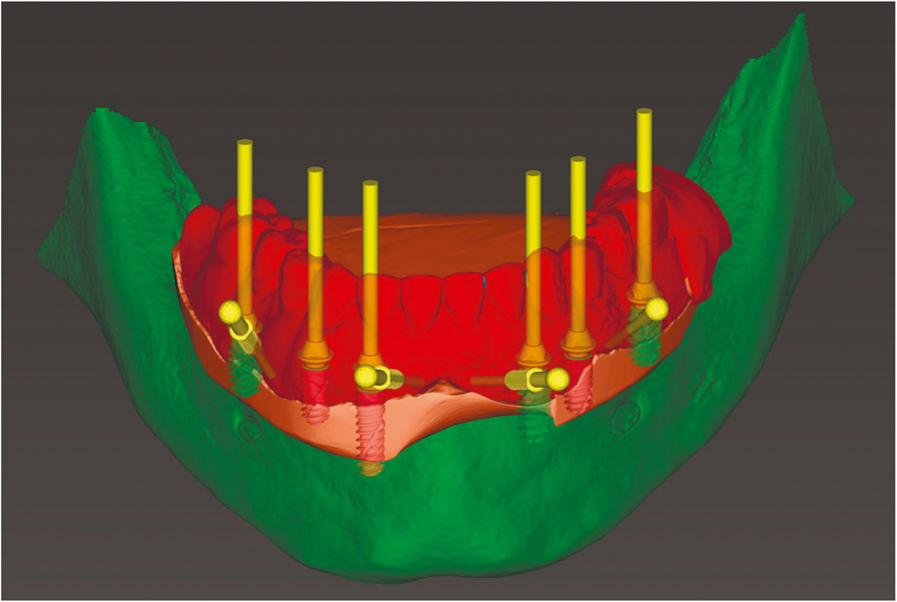
Matching DICOM and STL files, prosthetically driven implant surgical planning

**Fig. 3:**
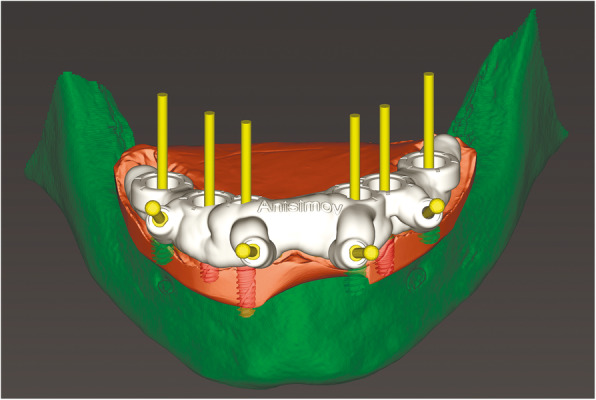
Computer-aided design of surgical templates for fixating pins

**Fig. 4:**
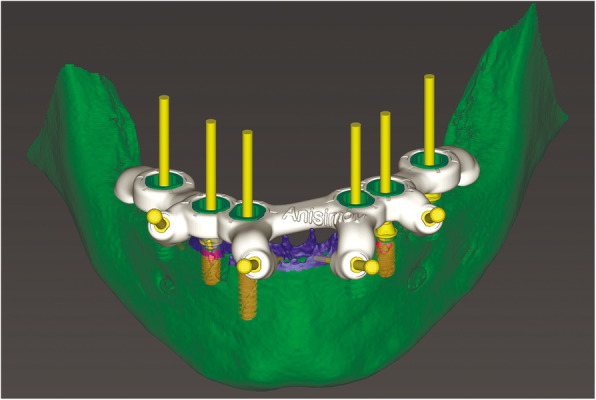
Computer-aided design of surgical templates for implant placement

**Fig. 5:**
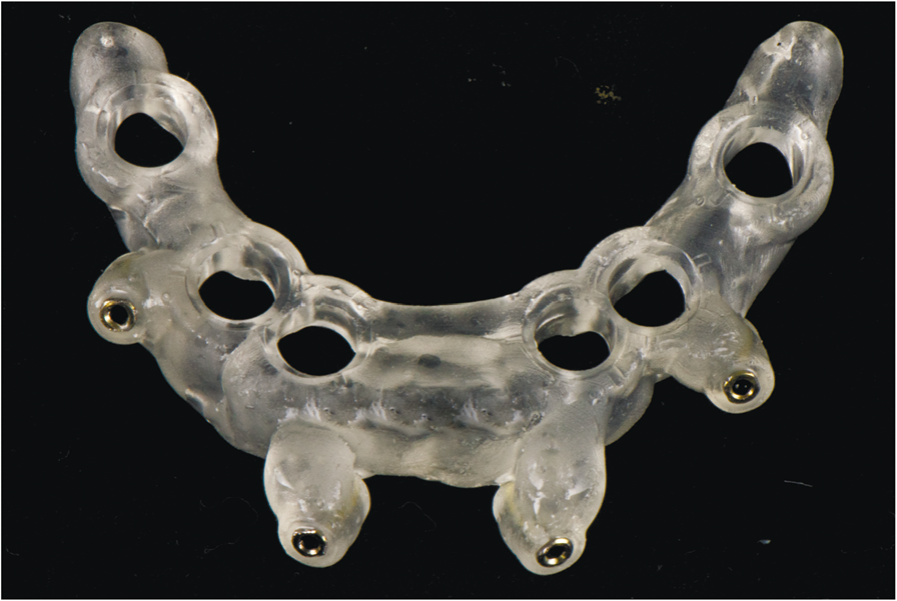
Computer-aided manufacturing of surgical templates for fixating pins

**Fig. 6:**
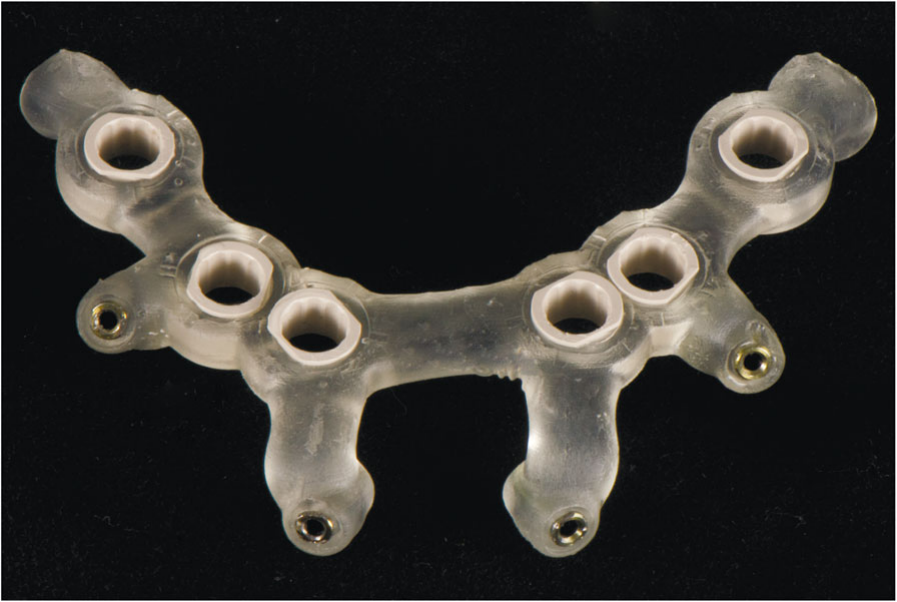
Computer-aided manufacturing of surgical templates for implant placement

**Fig. 7:**
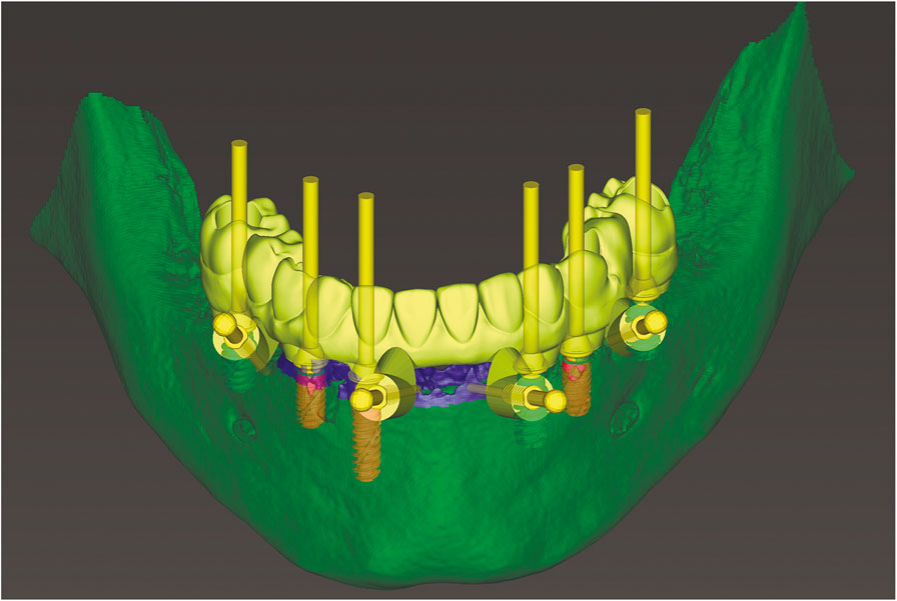
Computer-aided design of interim prosthesis

**Fig. 8:**
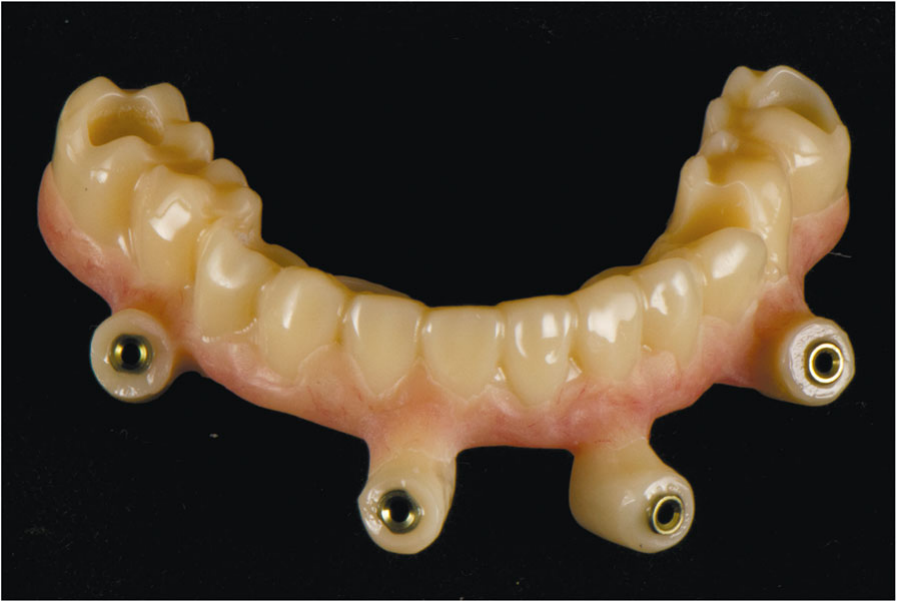
Computer-aided manufacturing of interim prosthesis with holes

**Fig. 9:**
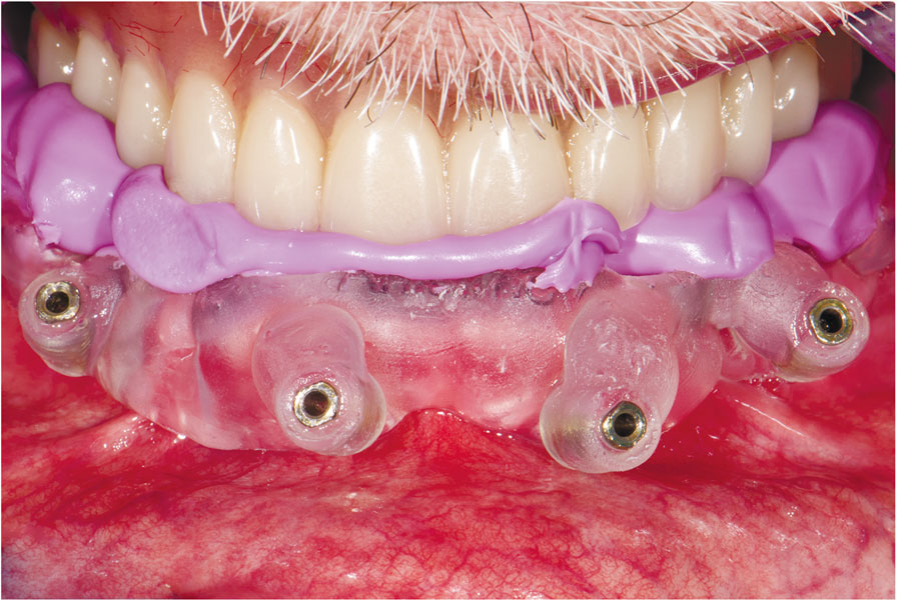
Template for fixating pins

**Fig. 10:**
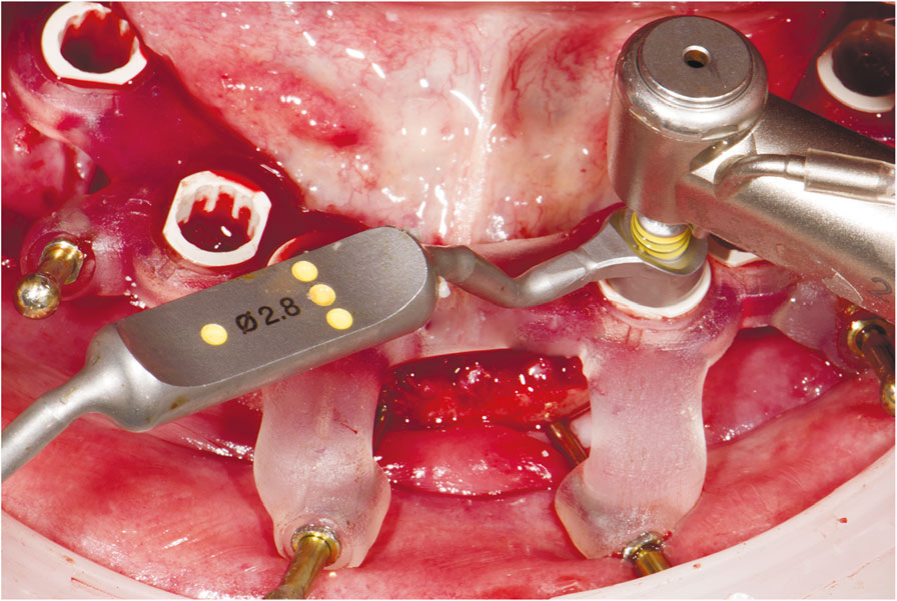
Implant osteotomies performed through a surgical template

**Fig. 11:**
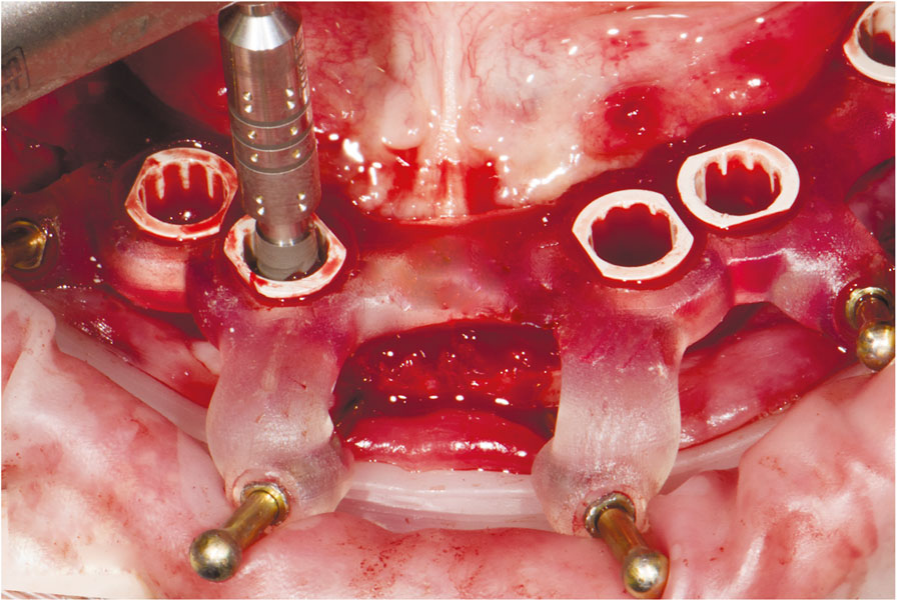
Guided implant placement

**Fig. 12:**
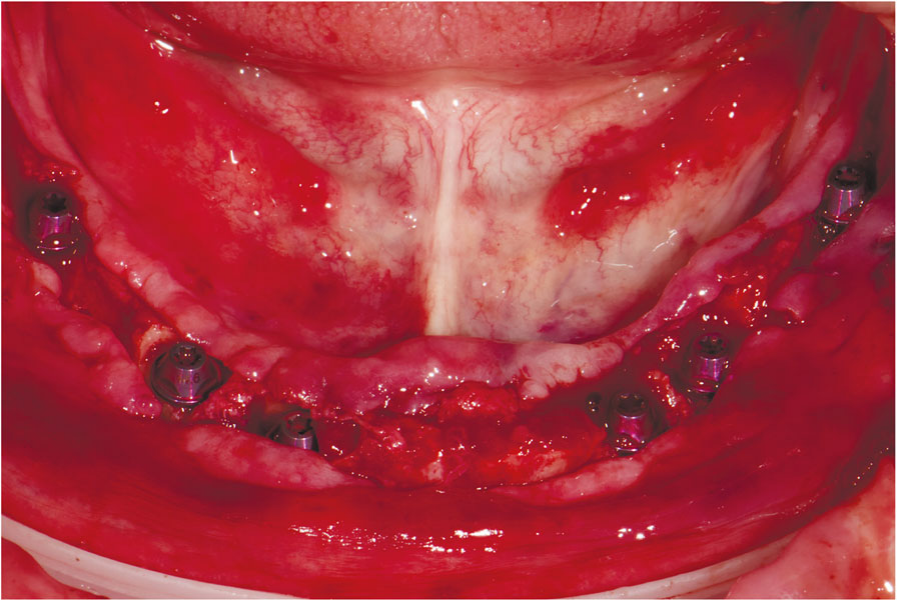
Screw-retained abutments fixation on implants

**Fig. 13:**
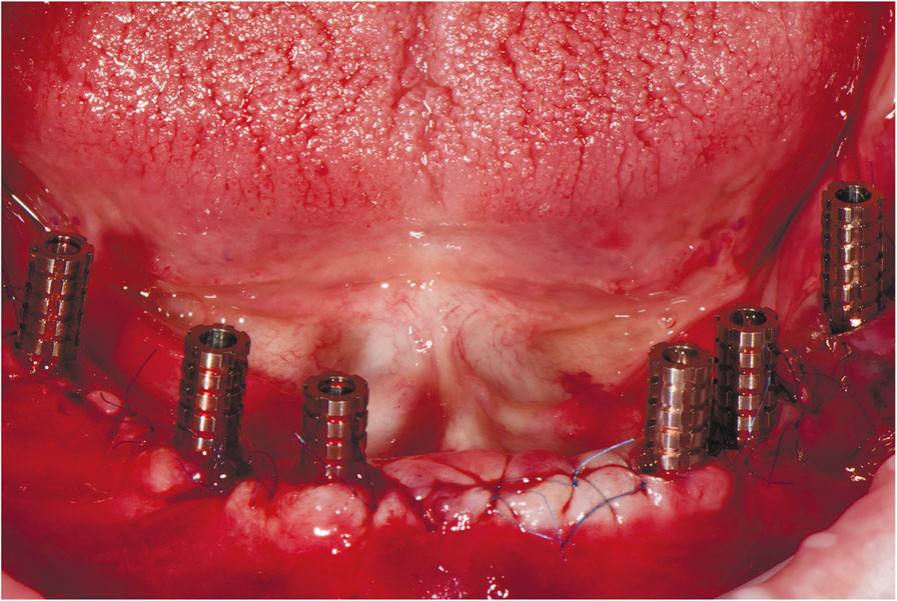
Interim titanium copings in situ fixed on screw-retained abutments

**Fig. 14:**
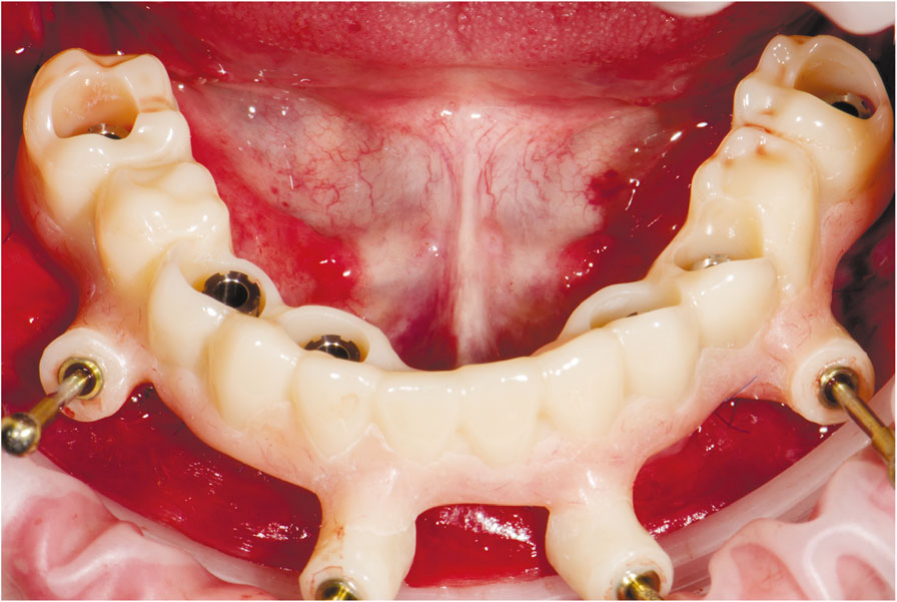
Interim prosthesis positioned and fixed with the same pins of the surgical guides

**Fig. 15:**
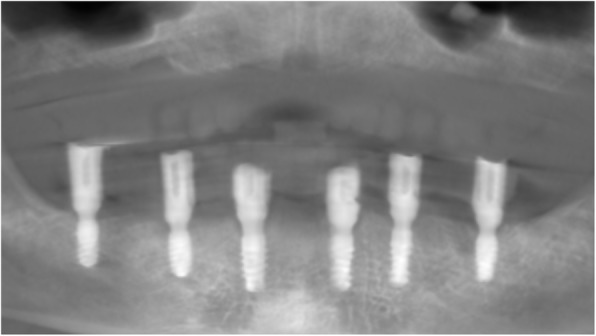
Panoramic radiograph after implant placement

At the first visit, each patient performed a panoramic radiograph to conduct initial treatment planning. All patients included in the study received new dentures with the intended occlusal vertical dimension, prepared using four teeth made of radiopaque resin to serve also as a radiographic template. The dentures were further relined before the acquisition of the cone-beam computer tomography (CBCT) scan for a precise transition of the actual soft tissue of the patient in the CAD (computer-aided design). A dual-scanning protocol was, then, implemented: a CBCT scan was performed at the patient wearing the radiographic template (Fig. 1) and separately at the radiographic template alone, in order to prosthetically orient implant positions and create a surgical template for computer-assisted implant placement. Conventional impressions of the radiographic template and the opposing arch were made with polyvinyl siloxane (Elite, Zhermack); furthermore, an intraoral occlusion index was recorded (Occlufast; Zhermack). Working casts (Type IV dental stone, Ultrarock) were poured and scanned by means of a laboratory scanner (7 Series; Dental Wings) to obtain STL (Standard Tessellation Language) files. CBCT data of the patients in DICOM (Digital Imaging and Communications in Medicine) format, STL and DICOM files of the radiographic templates were inserted and matched in a surgical planning software (coDiagnostiX, Dental Wings). All acquired data in STL format were superimposed, the implants and 4.6 mm diameter screw-retained abutments (SRA) were planned in prosthetically oriented positions (Fig. 2), and surgical templates with lateral fixation pin support and retention were designed for guided implant placement (Figs. 3 and 4). Surgical templates were then exported as STL files and 3D-printed (Straumann P 30+, Institut Straumann AG) (Figs. 5 and 6). The connection was established between dentists’ and dental technicians’ software with the “Synergy” function implemented in both software, which allowed them to work on the same patient in their software and introduce minor changes to the planning. The planning in surgical software was matched with the dental laboratory software Cares Visual (Institut Straumann AG) through CaseXchange data transfer channel. The dental technician designed the interim prostheses in accordance with the anatomy obtained by the STL files (Fig. 7); in all cases, the cantilever lengths were inferior to 15 mm [21]. The access holes were designed considering the planned SRA positions and the connectors of the prostheses were designed to be positioned with the same pins of the surgical templates. The prostheses were milled of PMMA-based resin blocks with subsequent pink resin veneering (Fig. 8) in a production facility (Createch). The basal surface of the prostheses was fabricated with an ovate shape, and a close contact with the mucosa was avoided to ensure cleanability. In none of the patients, there was a need to make a cast during prostheses fabrication. One hour before surgery, prophylactic antibiotics were given to patients: 2 g of amoxicillin (Zimox, Pfizer) or, in case of allergy, 500 mg of azithromycin (Zitromax, Pfizer). The protocol involved the use of two surgical templates: the first was mucosa or teeth-supported to drill the holes for fixation pins (Fig. 9); the second template was placed after raising a full-thickness flap and was supported by pins and soft or hard tissue distal support. When needed, minor bone reduction was performed. Osteotomies (Fig. 10) and implant placement (Fig. 11) were performed through the surgical template, and then tapered dental implants (BLX or BLT) with a sandblasted/long-grit/acid-etched active (SLActive) surface were inserted following proper manufacturers’ instructions and the surgical insertion protocol (Institut Straumann AG). Immediate loading was performed only when dental implants reached a minimum insertion torque of 35 Ncm. The SRA previously selected in the surgical planning software were tightened to the implants at 35 Ncm (Fig. 12) with a surgical motor with torque control (Implantmed, W&H). Interim titanium copings were, then, screwed on SRA abutments (Fig. 13) and the prefabricated prostheses were positioned using the same pins of the surgical guides (Fig. 14) and directly connected to titanium copings by means of an autopolymerizing resin (Acrytemp, Zhermack). The prostheses were, then, unscrewed and the flaps sutured with non-absorbable sutures (5.0 Prolene). Prostheses were polished, the connectors to the pins were removed, and they were delivered to the patients after occlusion checking, tightening the screws at 15 Ncm and sealing the screw access holes with a polytetrafluoroethylene (PTFE) tape and a flowable composite resin. A panoramic radiograph was taken immediately after implant placement (Fig. 15). Patients left the clinic with the interim fixed prostheses the same day of surgery.

Patients were instructed to rinse twice a day with an antiseptic mouthwash with chlorhexidine 0.2% (Curasept, Curaden Healthcare S.p.A) for 60 s starting for 10 days and to avoid tooth brushing for the first 2 weeks of healing. Furthermore, a soft diet was recommended, and ibuprofen 600 mg (Brufen, Abbott) was prescribed to be taken as needed. Medical check-ups were scheduled at 1 week and sutures were removed after 14 days. Then, patients were instructed to restart mechanical cleaning of the prosthetic surfaces and to use interdental brushes of different dimensions to clean the implant neck area and the apical component of the prostheses as proposed by Corbella et al. [22].

At the following appointments (1, 3, 6, and 12 months), specific oral hygiene instructions were given to patients, adapting interdental brush dimensions to the width of the gap between the mucosa and the prostheses due to soft tissue healing. The prostheses were firstly removed at the 3-month check-up to verify implants’ osseointegration. An implant in place at the end of the follow-up period was considered as a surviving implant.

A prosthesis in place at the end of the follow-up period was considered as a surviving prosthesis.

Prosthetic success was defined as a prosthesis that is stable and in good function and with the following characteristics: absence of abutment mobility, no implementation of corrective measures, or reparations to either prosthesis or abutment.

The following data were collected for each patient included in the study: number and position of dental implants, implant length and diameter, and opposing dentition (natural teeth, complete conventional denture, removable partial denture).

**Fig. 16:**
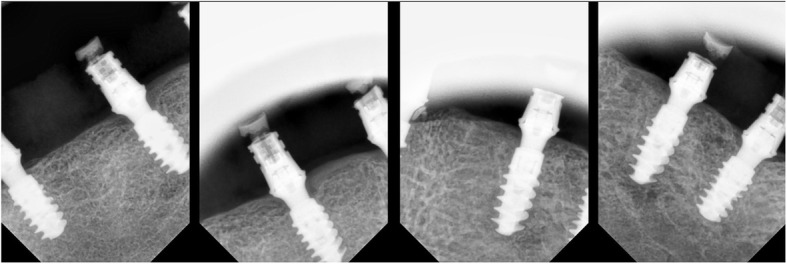
Periapical X-rays at 1 year

A specific analysis software (SOPRO Imaging, Acteon Group) was used to evaluate mean MBL levels on digital periapical X-rays acquired through an imaging plate scanner (PSPIX^2^®, Acteon Group, Norwich, UK) and taken by means of the parallel cone technique using a Rinn alignment system (XCP Centratore, Rinn, York, PA, USA). The bone level was digitally assessed for each implant mesially and distally by calculating the distance between the implant shoulder and the first visible bone contact. The implant length and width were used as references for calibration of measurements by two independent examiners who conducted the assessment. Measurements were taken after 1 year (Fig. 16).

A database was created with Excel (Microsoft). Descriptive statistics including mean ± SD values and percentage were calculated for each variable.

## Results

A total of ten patients were included in the study; they were either males (6) or females (4), with a mean age of 63.71 ± 14.55 years (range= 33–77 years). Eight patients were fully edentulous and were treated with conventional implant placement and immediate loading. Two patients were partially edentulous and underwent immediate implant placement: one of them had two teeth serving as attachments to a complete overdenture, the other patient had only one tooth to retain a removable partial denture.

A total of 55 dental implants (Table 1) and SRA (Table 2) were placed in ten patients by NM and PP.

**Table 1:** Distribution of implants according to length and diameter

Implant length	Implant diameters					
	3.3	3.75	4.1	4.5	5.5	6.5
6						3
8		3		19	2	
10	2	10		9		
12		2	3	2

**Table 2:** Distribution of screw-retained abutments according to gingiva height and diameter

	Diameters				
Gingiva height	1.5	2.5	3.5	4	4.5
0°	22	18	6	2	
17°		1			2
30°		2	2		

**Fig. 17:**
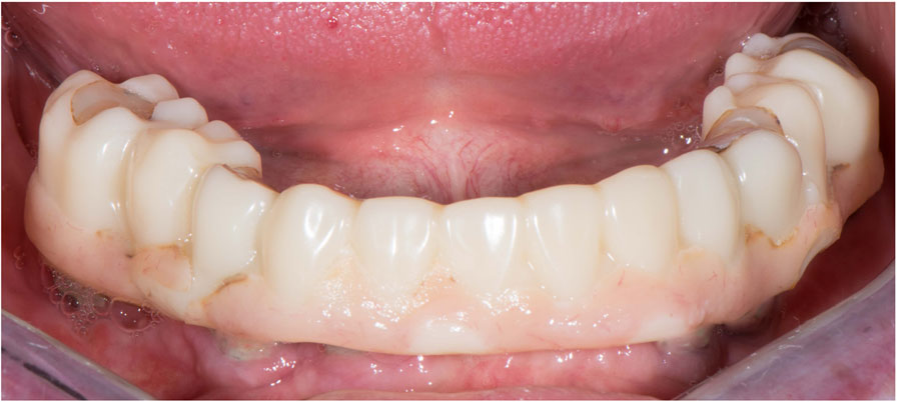
Interim prosthesis in situ at 1 year

Seven patients received six Straumann BLX SLActive (Institut Straumann AG) implants in the mandible with straight SRA. As opposing dentition, four of them had conventional complete denture, while two patients presented fixed dental prostheses supported by natural teeth and one patient had a removable partial denture as the antagonist. Two patients received four BLX implants in the mandible with two distal implants tilted with 17° angulated SRA in one patient and two distal implants tilted with 30° angulated SRA in the other patient. One of these patients had a conventional complete denture as an antagonist, the other had a fixed implant-supported prosthesis. One patient received five Straumann BLT SLActive (Institut Straumann AG) implants in the maxilla with two distal implants tilted with 30° angulated SRA, one distal implant with 17° angulated SRA, and two front implants placed axially with straight SRA. The patient had a removable partial denture as an opposing arch. No adverse reactions or wound healing complications were reported after implant placement. All the prostheses had 1 year of functional loading to simulate the long provisional phase (Fig. 17), with a prostheses survival rate of 100% and success rate of 70%. No screw loosening occurred at the third-month appointment. No fracture occurred during the whole period of the prostheses in situ. After 1 year, the mean MBL level was 0.37 ± 0.06 mm, while the implant survival rate was 98.18% (n=54/55).

All implants obtained high primary stability, with insertion torque values greater than 35 Ncm, and were immediately loaded, except for one BLT, which was not loaded and resulted in failure at the stage of the definitive prosthesis.

In three patients, the debonding of the interim prostheses to 3 temporary abutments occurred after implant osseointegration at the removal of the prostheses at 1 year and was successfully resolved without any further complication.

## Discussion

To the best of the authors’ knowledge, this is the first study evaluating prospectively the use of digitally prefabricated interim prostheses without cast in computer-assisted implant-supported full-arch rehabilitations.

All dental implants were placed with a s-CAIS protocol, raising full-thickness flaps to ensure the maximum keratinized mucosa preservation and using two surgical templates with the first guide mucosa- or tooth-supported to achieve the maximum accuracy for implant positioning [23, 24]. Implant quantity and position were decided on a patient-by-patient basis, considering bone availability, quality, anatomy, opposing dentition, and interocclusal recordings. In seven patients, bone quantity was enough for six implants to be placed, while in the remaining three patients the authors were limited by severe posterior mandibular atrophy and sinus anatomy. However, according to Polido et al. [25], there are no statistically significant differences in implant survival rates associated with the use of fewer than five implants when compared to five or more implants supporting a fixed dental prosthesis. While patients with four implants required computer-assisted implant placement due to the extremely difficult anatomy, subjects with six implants needed it for correct implant positioning for future definitive prostheses delivery. The authors suggest that this last consideration should apply to all edentulous patients and can also be viewed as a procedure of a high-quality implant-restoration planning.

With a complete computer-assisted protocol of implant placement and immediate loading, it is possible to fabricate a restoration cemented on a Titanium base before surgery [26]. However, according to several studies on the accuracy of s-CAIS [5–7] and the direct experience of the authors of analyzing the data with Treatment Evaluation Tool, a 100% accuracy in implant placement is not obtainable with s-CAIS. Thus, in full-arch rehabilitation, the provisional restorations cannot be cemented to abutments before surgery due to passivity concerns [27]. Therefore, in our study, interim prostheses were designed with wider access holes (5 mm diameter) around titanium copings in order to connect them with the resin directly in the mouth. The accuracy of fit of the interim restoration between temporary copings and access holes depends on the accuracy of the surgical template and the accuracy of the s-CAIS. In all patients, interim prostheses allowed the insertion of titanium copings without the need of access hole enlargement or adaptation. However, patients with tilted implants and 17 or 30 SRA appeared to be more technique sensitive, with the tilted implants that should be placed in the exact planned position to let the corresponding SRA axis match with the axis of the access holes in the prosthesis. This was planned in the surgical software by engraving rotation markers on the surgical templates that were used as a reference when inserting the implant with the guided adapter. Different studies have demonstrated that patients use provisional prostheses for several months, until they receive the definitive rehabilitation [28, 29]. Therefore, obtaining durable interim prostheses is mandatory in order to complete a successful rehabilitation and to allow a proper osseointegration for implants inserted. The evidence related to specific oral hygiene protocols for the implant-supported full arch is still limited: recently, Maeda et al. [30] reported that electric toothbrushes are more effective than manual toothbrushes for plaque removal in these patients. However, there are no data on the influence of the prosthetic design on cleanability.

In the present study, milled PMMA restorations proved to be durable enough during the long provisional phase. Hence, the superior flexural strength properties of PMMA considerably lowers the risk of fracture compared to conventional dentures converted in implant-supported interim prostheses [31]. Furthermore, a surface roughness below the plaque accumulation threshold has been reported for PMMA interim restorations in an in vitro study [32], therefore suggesting a lower risk of bacterial contamination for the prosthetic structure. The main limitation of this study is the small sample enrolled; furthermore, opposing dentition characteristics might have influenced the outcomes of this study, and hence, five patients had complete conventional dentures, generally associated with a lower incidence of prosthetic complications [33]. However, to the best of the authors’ knowledge, the other studies in literature reporting data on prefabricated prostheses without cast present just case reports or description of clinical techniques [19, 20]; therefore, there are no studies to which our findings can be directly compared.

Future research should be orientated in conducting further randomized controlled clinical trials, with a larger sample, to evaluate the accuracy of full-arch interim prostheses fabricated with the digital technique without cast compared to conventional workflow.

## Conclusions

Within the limitations of the present study, the authors concluded that digitally prefabricated provisional prostheses for full-arch immediate loading with s-CAIS could be a valid alternative treatment modality. Milled PMMA restorations proved to be durable enough during the long provisional phase, without major prosthetic complications.

## Data Availability

The datasets used and/or analyzed during the current study are available from the corresponding author on reasonable request.

## Abbreviations

s-CAIS: Static computer-aided implant surgery
PMMA: Polymethyl methacrylate
CAD/CAM: Computer-aided-design and computer-aided-manufacturing
CBCT: Cone-beam computer tomography
STL: Standard Tessellation Language
DICOM: Digital Imaging and Communications in Medicine
SRA: Screw-retained abutments
SLActive: Sandblasted/long-grit/acid-etched active
PTFE: Polytetrafluoroethylene
MBL: Marginal bone loss
